# Clinical Outcomes and Microbiological Characteristics of Sequence Type 11 *Klebsiella pneumoniae* Infection

**DOI:** 10.3389/fmed.2022.889020

**Published:** 2022-05-16

**Authors:** Ping Yang, Zhenchao Wu, Chao Liu, Jiajia Zheng, Nan Wu, Zhangli Wu, Juan Yi, Ming Lu, Ning Shen

**Affiliations:** ^1^Institute of Medical Technology, Peking University Health Science Center, Beijing, China; ^2^Department of Pulmonary and Critical Care Medicine, Peking University Third Hospital, Beijing, China; ^3^Department of Infectious Diseases, Peking University Third Hospital, Beijing, China; ^4^Laboratory of Clinical Microbiology, Peking University Third Hospital, Beijing, China

**Keywords:** *Klebsiella pneumoniae*, ST11, risk factor, virulence, multidrug resistance

## Abstract

**Background:**

Sequence type 11 (ST11) *Klebsiella pneumoniae* (Kp) is highly prevalent in China and is a typical sequence type among KPC-producing isolates. This study aimed to evaluate the clinical outcomes and microbiological features of ST11 Kp infections.

**Methods:**

A retrospective cohort study was conducted at Peking University Third Hospital from January 2017 to March 2021. Clinical data were collected from medical records. Antimicrobial susceptibility testing and string tests were performed. Whole-genome sequencing was used to analyze the capsular serotypes, detect virulence-associated genes, and perform multilocus sequence typing. The risk of all-cause mortality in ST11 Kp-infected patients was compared to that in non-ST11 Kp-infected patients.

**Results:**

From 139 patients infected with Kp, 49 ST11 Kp (35.3%) strains were isolated. The Charlson comorbidity index in the ST11 group was higher than that in the non-ST11 group (3.94 ± 1.59 vs. 2.41 ± 1.54, *P* = 0.001). A greater number of ST11 Kp-infected patients required ICU admission (46.9 vs. 16.7%, *P* < 0.001) and mechanical ventilation (28.6 vs. 10.0%, *P* = 0.005). All ST11 isolates presented a multidrug-resistant (MDR) phenotype, and twenty-nine (59.2%) hypervirulent Kp (hvKp) were identified. Twenty-four ST11 strains presented with hypermucoviscosity. The presence of capsular types K47 and K64 was frequent in the ST11 Kp strains (*P* < 0.001). The key virulence-associated genes *rmpA, rmpA2, iucA, iroB*, and *peg344* were present in 26.5, 42.9, 59.2, 0, and 26.5% of the isolates, respectively, in the ST11 group. Twenty-one ST11 isolates harbored the combination of *iucA*+*rmpA2*. The 30-day mortality rate and sequential organ failure assessment (SOFA) score were significantly higher in ST11 Kp-infected patients than in non-ST11 Kp-infected patients (*P* < 0.01). ST11 Kp infection appeared to be an independent risk factor for mortality in ST11 Kp-infected patients.

**Conclusions:**

A high prevalence of the ST11 clone was found in the hospital, which accounted for elevated antimicrobial resistance and exhibited great molecularly inferred virulence. Patients with ST11 Kp infection had a tendency toward increased 30-day mortality and SOFA scores. ST11 Kp infection was an independent risk factor for mortality, suggesting that enhanced surveillance and management are essential.

## Introduction

*Klebsiella pneumoniae* (Kp), an important community-acquired and nosocomial gram-negative bacterial pathogen, causes fatal infections, including pneumonia, urinary tract infection, pyogenic liver abscess, bacteremia and so on (1). Two distinct pathotypes are currently circulating: hypervirulent *Klebsiella pneumoniae* (hvKp) and classical *Klebsiella pneumoniae* (cKp), each of which poses great challenges in clinical practice (2–5). cKp infection mainly occurs in immunocompromised hosts and patients with physiological barrier breakdown in the medical environment. cKp has the ability to acquire various antibiotic resistance genes and rapidly become resistant to all available antibiotics, which is a serious threat to public health. The worldwide spread of multidrug-resistant (MDR) cKp is mainly driven by a special clone termed sequence type 11 (ST11) (6, 7). In China, ST11 Kp is endemic, and most KPC-producing isolates are typically of this sequence type (8). In stark contrast, hvKp infection is mainly acquired by healthy individuals of any age in the community and is highly associated with aggressive invasive infections in hospitals, such as bacteremia and pyogenic liver abscess (4, 9, 10). The previous definition of hvKp was determined by the hypermucoviscosity phenotype (string test showing a positive result) (11).

In fact, not all hvKp isolates possess the hypermucoviscosity phenotype, as confirmed by *in vitro* and *in vivo* studies (3, 5, 12–14). Studies have demonstrated that genetic traits perform better than hypermucoviscosity as a marker for differentiating hvKp and cKp (14, 15). Five key virulence-associated genes, *iucA, iroB, peg-344, rmpA* and *rmpA2*, showed higher diagnostic accuracy for defining hvKp than the hypermucoviscosity phenotype and other virulence-associated genes (15).

ST11, previously identified as cKp, commonly presents with lower virulence (16, 17). However, Gu et al. (18) first demonstrated a fatal outbreak caused by ST11 carbapenem-resistant (CR) hvKp in the ICU. The acquisition of pVir-CR-hvKp4 contributed to the convergence of carbapenem resistance and hypervirulence (18). In addition, the emergence of hybrid conjugative virulence plasmids triggered MDR hvKp formation (19, 20).

Contrary to common belief, the phenomenon of convergence of carbapenem resistance and hypervirulence in Kp is increasing in frequency, which may lead to worse clinical outcomes (18, 19). Few studies have focused on the outcomes and microbiological characteristics of ST11 Kp strains. Liu et al. (21) revealed that the 3-year survival rate of ST11 Kp-infected patients was 73.68%. Another study reported that the in-hospital mortality in the ST11 group was 36.7% (22). However, these previous studies on the mortality of ST11 Kp infection compared to that of non-ST11 infection were controversial. Therefore, we conducted a retrospective study to analyze the clinical outcomes, antibiotic resistance, virulence, and all-cause mortality risk of ST11 Kp infection. We found that ST11 Kp isolates accounted for elevated antimicrobial resistance and exhibited great molecularly inferred virulence. ST11 Kp infection was an independent risk factor for mortality and an increased sequential organ failure assessment (SOFA) score.

## Materials and Methods

### Enrolled Patients

A retrospective cohort study was conducted on Kp culture-positive patients enrolled from January 2017 to March 2021 at Peking University Third Hospital. Clinical data and patient information were obtained from medical records, which included basic demographics, medical history, usage of invasive devices, infection site, infection type, blood examination results, ICU admission, and mechanical ventilation after Kp infection. The Charlson comorbidity index (CCI) was calculated based on the medical history.

The main inclusion criteria were as follows: (1) age ≥ 18 years old and (2) Kp was cultured positively and was associated with clinical infectious manifestations at the same time. The exclusion criteria included the following: (1) the bacterial strain not viable after storage and (2) duplicate isolates from the same patient within 3 months. The primary outcome was survival and all-cause mortality at 30 days after Kp infection, and the secondary outcome was the SOFA score.

Hospital-acquired infection and community-acquired infection were defined as previously described (4). The definition of metastatic infection was based on the clinical diagnosis (the presence of > 1 infection site) in the same patient (23).

This study protocol was approved by the Peking University Third Hospital Medical Science Research Ethics Committee (M2021545).

### Strain Identification and Antimicrobial Susceptibility Profiling

These specimens were from the respiratory system, urine, blood, drainage fluid, and other body sites. The standardized isolation, culture, and identification were conducted in the Department of Clinical Microbiology. All strains were stored at −80°C.

Strain identification and antimicrobial susceptibility testing (AST) were performed by a Vitek 2 system (bioMérieux, Marcy-l'Étoile, France). If necessary, we also applied the disk diffusion method. The AST results were interpreted according to the 2020 Clinical and Laboratory Standards Institute (CLSI) guidelines (24). The following antimicrobial agents were tested: piperacillin/tazobactam, cefoperazone/sulbactam, ceftazidime, cefepime, imipenem, meropenem, levofloxacin, amikacin, minocycline, and trimethoprim/sulfamethoxazole. The definition of an MDR strain was resistance to three or more different antimicrobial categories (25). Carbapenem-resistant *Klebsiella pneumoniae* (CRKP) was defined based on resistance to imipenem or meropenem.

### DNA Extraction and Whole-Genome Sequencing

The DNA of all the isolated Kp strains was extracted by using a GenePure Pro Automatic Nucleic Acid Purification System (NPA-32P, Bioer Technology, Hangzhou, Zhejiang, China) and MagaBio Bacterium DNA Fast Purification Kit (BSC45S1E, Bioer Technology, Hangzhou, Zhejiang, China). Each Kp sample was fully mixed with 180 μL of TET buffer (with added lysozyme) and incubated at 37°C for 30~60 min. Two microliters of RNase A (RT405-02, TIANGEN, Beijing, China) was added, and the samples were shaken for 15 s and incubated at 15–25°C for 5 min. Next, each prepared sample and 20 μl of Proteinase K were transferred into kit columns 1 and 7, and then plugged in 8-strip tip and ran the machine program. DNA concentration and purity were evaluated by a NanoDrop (ThermoFisher, Waltham, America).

All strains were sequenced using the Illumina HiSeq 2500 platform by constructing paired-end libraries to obtain 150 bp reads. The clean data were obtained using FastQC and assembled using SPAdes (v.3.13) with the default parameters.

### Bioinformation Analysis and Phenotypic Features

Whole-genome sequencing was used to analyze the capsular serotypes, identify virulence-associated genes, and perform multilocus sequence typing (MLST). Raw data were filtered to remove low-quality reads and then assembled using SPAdes (v.3.13). The definition of ST11 Kp was based on MLST using MLST (v.2.0) (Center for Genomic Epidemiology). Capsular types were analyzed using Kleborate software (v.0.3.0). Resistance and virulence genes were annotated by comparison with relevant databases (ResFinder, Virulence Factor Database) using BLAST software (v.2.2.18). HvKp is defined based on the combination of *peg-344, iroB, iucA, rmpA*, or *rmpA2* positivity (15).

Sequencing reads were mapped to the *K. pneumoniae* HS11286 using bowtie 2 v2.2.8 and single nucleotide polymorphisms (SNPs) were identified by using Samtools v1.9 and combined according to the reference genome (SGH-10) using the iSNV-calling pipeline (https://github.com/generality/iSNV-calling). High-quality SNPs (more than 5 reads of mapping quality > 20) were retained. Regions of recombination were detected by Gubbins (26), and the polymorphic sites located in recombination regions were removed. The concatenated sequences of filtered polymorphic sites conserved in all genomes (core genome SNPs, cgSNPs) were used to perform phylogenetic analysis using the maximum likelihood method by iqTree2.1.2 (27).

The hypermucoviscous phenotype was determined by the string test as described previously (2). All isolates were inoculated onto Columbia agar with sheep blood (PB0123A, OXOID, Beijing, China) and incubated at 37°C overnight. The string test was considered positive when a bacteriology inoculation loop was able to generate a viscous string > 5 mm in length by touching and pulling a single colony upward.

### Statistical Analysis

Data analysis was performed using SPSS software (v.25.0). Measurement data were assessed as the mean ± standard deviation, and count data are reported as percentages. We used the *T* test and Wilcoxon test for the analysis of continuous variables. We performed the χ^2^ or Fisher's exact test for categorical variables. All tests were 2-tailed. A *P*-value < 0.05 was considered statistically significant. The all-cause mortality within 30 days between the two groups was estimated using a Kaplan–Meier curve and log-rank tests. Univariate logistic regression analyses were performed to identify the risk factors associated with death. A multivariable logistic regression analysis was conducted for independent risk factors for death (the variables with *P* < 0.05 were included). Univariate linear regression analyses were performed to identify the factors associated with death. A multivariable linear regression analysis was conducted for independent risk factors for elevated SOFA scores (the variables with *P* < 0.05 were included).

## Results

### Clinical Characteristics of ST11 Kp Infection

Isolates from 139 Kp infection cases were collected from January 2017 to March 2021. Among these isolates, 49 strains (35.3%, 49/139) were identified as ST11, which was the most prevalent sequence type in this study. Non-ST11 strains (64.7%, 90/139) mainly included ST23 (18.9%, 17/90), ST15 (8.9%, 8/90), ST86 (6.7%, 6/90) and so on. The median age of patients with ST11 Kp infections was 80.04 ± 12.41 years, and 29 patients (59.2%) were male. Most ST11 Kp strains were isolated from patients in the emergency department and ICU (55.1 and 26.5%, respectively), followed by the geriatric department (10.2%) (Supplementary Figure 1). Compared with those in the non-ST11 group, more patients in the ST11 group had cardiovascular disease (93.9 vs. 64.4%, *P* < 0.001), cerebrovascular disease (63.3 vs. 36.7%, *P* = 0.003), and urinary disease (51.0 vs. 28.9%, *P* = 0.010). Furthermore, the CCI was higher in the ST11 group (3.94 ± 1.59 vs. 2.41 ± 1.54, *P* = 0.001). In addition, the ST11 group showed significantly more antibiotic exposure within the previous 90 days (100.0 vs. 60.0%, *P* < 0.001). A significant number of patients with invasive catheters were infected by ST11 Kp isolates (100.0 vs. 55.6%, *P* < 0.001), which included central intravenous catheters (67.3 vs. 26.0%, *P* < 0.001), urinary catheters (91.8 vs. 70.0%, *P* = 0.006), endotracheal tubes (32.7 vs. 14.0%, *P* = 0.028) and gastrostomy tubes (89.8 vs. 60.0%, *P* = 0.001). In this study, the most common ST11 Kp infection sites were the respiratory system (65.3%), followed by urine (16.3%). Fewer patients suffered from bloodstream infection in the ST11 group than in the non-ST11 group (6.1 vs. 18.9%, *P* = 0.040), whereas there was no significant difference among other sites between the two groups. Compared to non-ST11 Kp strains, ST11 Kp strains were more closely related to hospital-acquired infections (100.0 vs. 74.4%, *P* < 0.001). In contrast, more community-acquired infections occurred in non-ST11 isolates (0 vs. 25.6%, *P* < 0.001). Furthermore, blood testing indicators (red blood cell count, hemoglobin, and albumin) of patients with ST11 Kp infection were significantly lower than those of the non-ST11 group (*P* < 0.01). However, patients with ST11 Kp infection had a higher hematocrit (*P* = 0.013). In addition, a significant number of patients with ST11 Kp infection required ICU admission (46.9 vs. 16.7%, *P* < 0.001) and mechanical ventilation after Kp detection (28.6 vs. 10.0%, *P* = 0.005) (Table 1).

**Table 1:** Clinical Characteristics of ST11 vs. Non-ST11 Kp.

**Clinical characteristics**	**ST11 (*n* = 49)**	**Non-ST11 (*n* = 90)**	***P*** **-value**
**Basic demographics**			
Age	80.04 ± 12.41	68.66 ± 19.27	0.005
Male	29 (59.2%)	61 (67.8%)	0.311
**Medical history**			
Diabetes	20 (40.8%)	30 (33.3%)	0.380
Pulmonary disease	18 (36.7%)	22 (24.4%)	0.126
Cardiovascular disease	46 (93.9%)	58 (64.4%)	0.000
Cerebrovascular disease	31 (63.3%)	33 (36.7%)	0.003
Digestive disease	15 (30.6%)	34 (37.8%)	0.398
Urinary disease	25 (51.0%)	26 (28.9%)	0.010
Cancer	10 (20.4%)	11 (12.2%)	0.198
CCI^a^	3.94 ± 1.59	2.41 ± 1.54	0.001
Surgery within 3 months	3 (6.1%)	8 (8.9%)	0.804
Antibiotic exposure within 90 days	49 (100.0%)	54 (60.0%)	0.000
Usage of invasive catheters	49 (100.0%)	50 (55.6%)	0.000
Central intravenous catheter	33 (67.3%)	13 (26.0%)	0.000
Urinary catheter	45 (91.8%)	35 (70.0%)	0.006
Endotracheal tube	16 (32.7%)	7 (14.0%)	0.028
Gastrostomy tube	44 (89.8%)	30 (60.0%)	0.001
Drainage tube	13 (26.5%)	11 (22.0%)	0.599
Metastatic infection	13 (26.5%)	13 (14.4%)	0.081
**Infection site**			
Respiratory tract	32 (65.3%)	47 (52.2%)	0.137
Urinary tract	8 (16.3%)	12 (13.3%)	0.631
Blood	3 (6.1%)	17 (18.9%)	0.040
Drainage	1 (2.0%)	3 (3.3%)	1.000
Other	5 (10.2%)	11 (12.2%)	0.722
**Infection type**			
Hospital-acquired infection	49 (100.0%)	67 (74.4%)	0.000
Community-acquired infection	0 (0.0%)	23 (25.6%)	0.000
**Laboratory examination result**			
Red blood cell count	3.03 ± 0.80	3.64 ± 0.93	0.000
Hemoglobin	94.47 ± 23.82	110.18 ± 25.65	0.001
White blood cell count	10.03 ± 4.90	10.48 ± 5.17	0.611
Platelet count	180.92 ± 128.76	218.12 ± 98.02	0.082
NEU%^b^	77.87 ± 15.68	78.60 ± 15.77	0.986
Total protein	61.81 ± 7.73	63.87 ± 10.23	0.202
Albumin	30.78 ± 3.96	32.84 ± 6.00	0.018
Hematocrit	0.73 ± 3.03	0.33 ± 0.08	0.013
Vasoactive drug use after Kp detection	10 (20.4%)	10 (11.1%)	0.136
Admitted in the ICU^c^	23 (46.9%)	15 (16.7%)	0.000
Mechanical ventilation after Kp detection	14 (28.6%)	9 (10.0%)	0.005
SOFA^d^	6.31 ± 6.04	2.47 ± 2.97	0.000
30-day mortality	18 (38.3%)	11 (12.5%)	0.001

a *CCI, Charlson comorbidity index*.

b *NEU%, Neutrophil percentage*.

c *Patients infected with Kp were then transferred to the ICU*.

d *SOFA, Sequential organ failure assessment*.

Subgroup analysis was performed according to cKp and hvKp infection. The clinical characteristics of ST11 Kp infection in the hvKp and cKp subgroups were mostly similar to the above results. Notably, more metastatic infection occurred with ST11 Kp strains of the hvKp subgroup (34.5 vs. 10.9%, *P* = 0.013) (Supplementary Table 2). In the cKp subgroup, more patients suffered from respiratory system infection in the ST11 group than in the non-ST11 group (75.0 vs. 47.7%, *P* = 0.041) (Supplementary Table 2). Additionally, in the ST11 group, the ICU admission rate was higher in patients with cKp infection than in those with hvKp infection (34.5 vs. 65.0%, *P* = 0.035), while the other clinical characteristics showed no significant differences (Supplementary Table 3).

### Microbiological Characteristics of ST11 Kp Infection

All the ST11 Kp isolates (100.0%) were MDR, while only 28 of 90 non-ST11 (31.1%) Kp strains were MDR. Moreover, CRKP in the ST11 group and non-ST11 group accounted for 100 and 14.4%, respectively. Notably, all 49 ST11 Kp strains (100.0%) were resistant to piperacillin/tazobactam, cefoperazone/sulbactam, ceftazidime, cefepime, imipenem, and meropenem. Significantly, the rates of resistance to all antibiotics in the non-ST11 group were <50%. However, except for minocycline and trimethoprim/sulfamethoxazole, the rates of resistance to most antibiotics were approximately 100% in ST11 Kp isolates. Additionally, for the antibiotics commonly used in clinical practice, such as β-lactamase inhibitors and carbapenems, the resistance rate of the ST11 group was significantly higher than that of the non-ST11 group (*P* < 0.001) (Table 2, Supplementary Table 1).

**Table 2:** Antibiotic resistance patterns of ST11 Kp.

**Antibiotic agent**	**ST11**	**Non-ST11**	***P*** **-value**
MDR^a^	49 (100.0%)	28 (31.1%)	0.000
CR^b^	49 (100.0%)	13 (14.4%)	0.000
Piperacillin/tazobactam (TZP)	49 (100.0%)	15 (16.7%)	0.000
Cefoperazone/sulbactam (CSL)	49 (100.0%)	14 (15.6%)	0.000
Ceftazidime (CAZ)	49 (100.0%)	22 (24.4%)	0.000
Cefepime (FEP)	49 (100.0%)	24 (26.7%)	0.000
Imipenem (IPM)	49 (100.0%)	12 (13.3%)	0.000
Meropenem (MEM)	49 (100.0%)	13 (14.4%)	0.000
Levofloxacin (LVX)	48 (98.0%)	25 (27.8%)	0.000
Amikacin (AMK)	42 (85.7%)	5 (5.6%)	0.000
Minocycline (MNO)	23 (46.9%)	19 (21.1%)	0.002
Trimethoprim/sulfamethoxazole (SXT)	22 (44.9%)	18 (20.0%)	0.002

a *MDR, multidrug resistant*.

b *CR, carbapenem resistant*.

**Figure 1:**
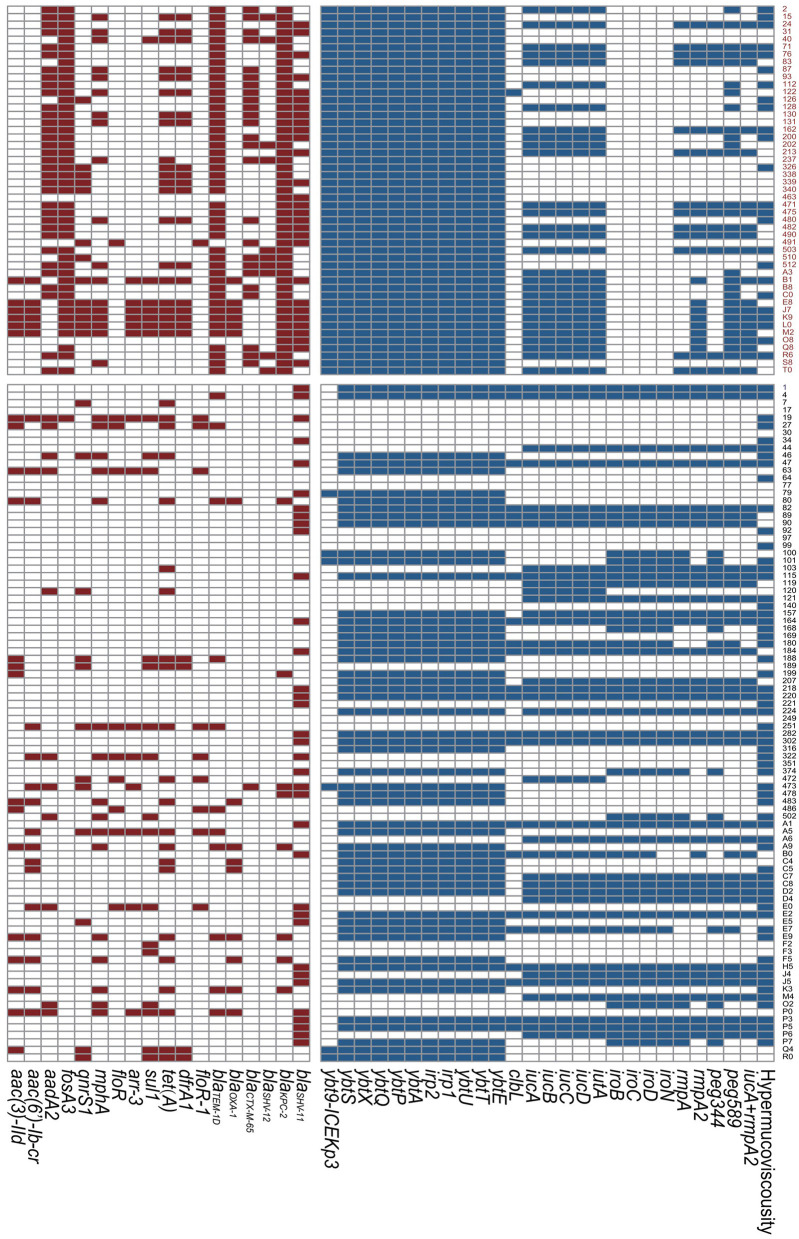
Resistance genes and virulence genes of *Klebsiella pneumoniae* strains. The colored blocks represent existence of genes. Red, ST11 group; Black, Non-ST11 group.

In terms of resistance genes, all the ST11 Kp strains harbored the beta-lactamase gene *bla*_*KPC*−2_. Additionally, a large number of ST11 isolates presented the beta-lactamase gene *bla*_*TEM*−1*D*_ (93.9%) and the fosfomycin resistance gene *fosA3* (91.8%). Over half of the ST11 isolates possessed the aminoglycoside resistance gene *aadA2* and the beta-lactamase genes *bla*_*CTX*−*M*−65_ and *bla*_*SHV*−11_, which were significantly more frequently detected than in the non-ST11 group (*P* < 0.001, *P* < 0.001 and *P* = 0.022, respectively). There was a strong tendency for the presence of *K47* (53.1 vs. 1.1%, *P* < 0.001) and *K64* (42.9 vs. 1.1%, *P* < 0.001) in ST11 Kp strains. In contrast, there was a tendency for the presence of *K1* (0 vs. 20.0%, *P* < 0.001) and *K2* (0 vs. 15.6%, *P* = 0.002) in non-ST11 Kp isolates. Twenty-four ST11 strains showed a hypermucoviscous phenotype, which was a significantly lower number than that in the non-ST11 group (49.0 vs. 68.9%, *P* = 0.021). The virulence-associated genes *rmpA, rmpA2, iucA, iroB*, and *peg344* were present in 26.5, 42.9, 59.2, 0, and 26.5% of the isolates in the ST11 group, respectively. Notably, all the ST11 Kp isolates harbored the siderophore yersiniabactin genes. Additionally, the *ybt-ICEKp3* cluster was highly clustered in the ST11 group (100.0 vs. 6.7%, *P* < 0.001). *peg589*, which is related to a poor prognosis in an animal model, was highly associated with the ST11 group (61.2 vs. 41.1%, *P* = 0.023). Importantly, twenty-nine (59.2%) ST11 Kp strains were MDR hvKp. Additionally, twenty-one ST11 isolates harbored the combination of *iucA*+*rmpA2*, which showed no significant difference in both groups (*P* = 0.649) (Figure 1, Supplementary Table 4).

**Figure 2:**
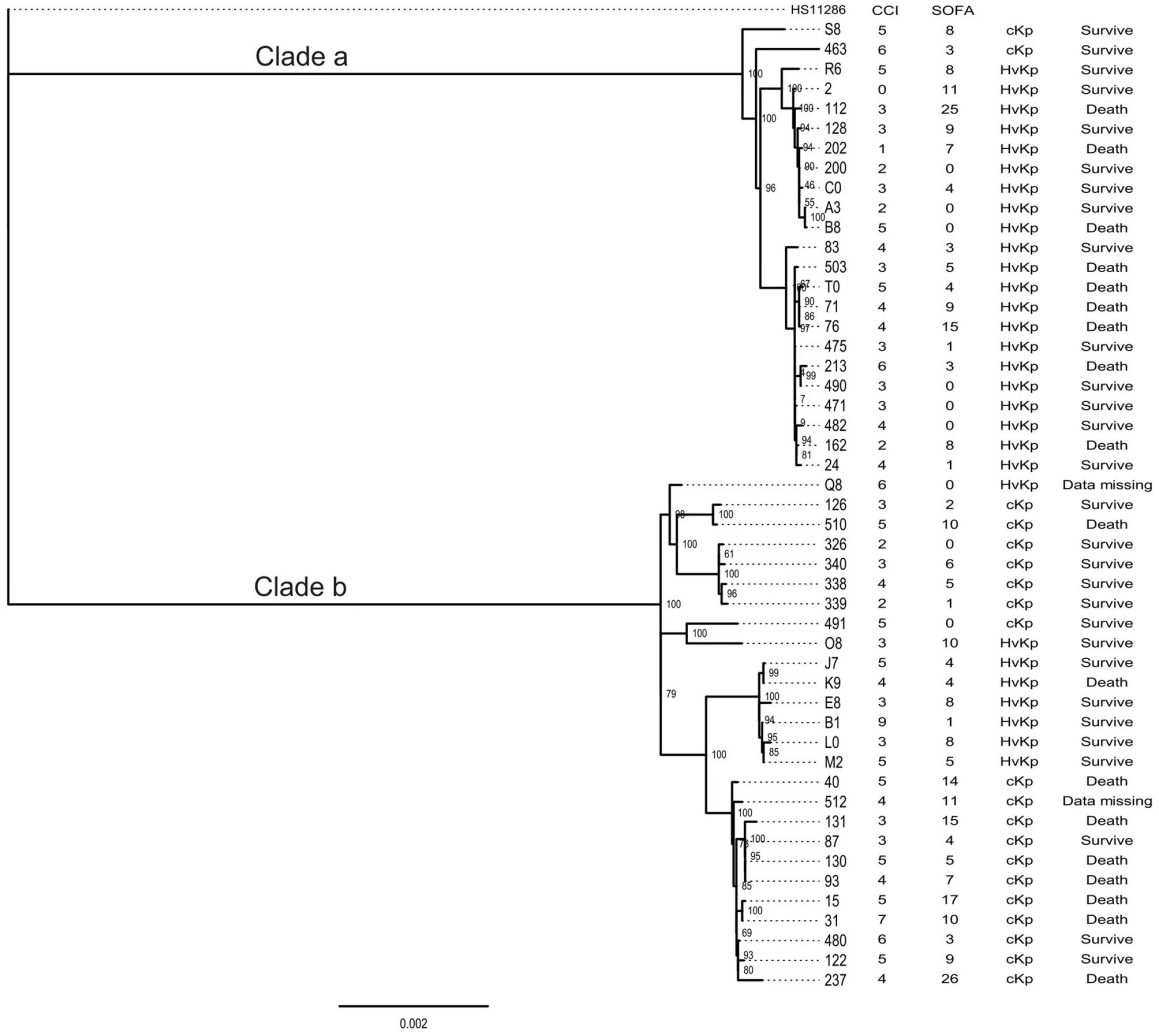
The phylogenetic tree of ST11 strains isolated in this study.

Among all the ST11 Kp strains, two distinguished clades were identified by the phylogenetic analysis. Clade a contained 23 strains, and clade b comprised 26 strains. Notably, a great number of hvKp belonged to the clade a (21/29). Besides, two clades had the same 30-day mortality (Figure 2).

### Risk Factors for 30-Day Mortality in Patients With Kp Infection

**Figure 3:**
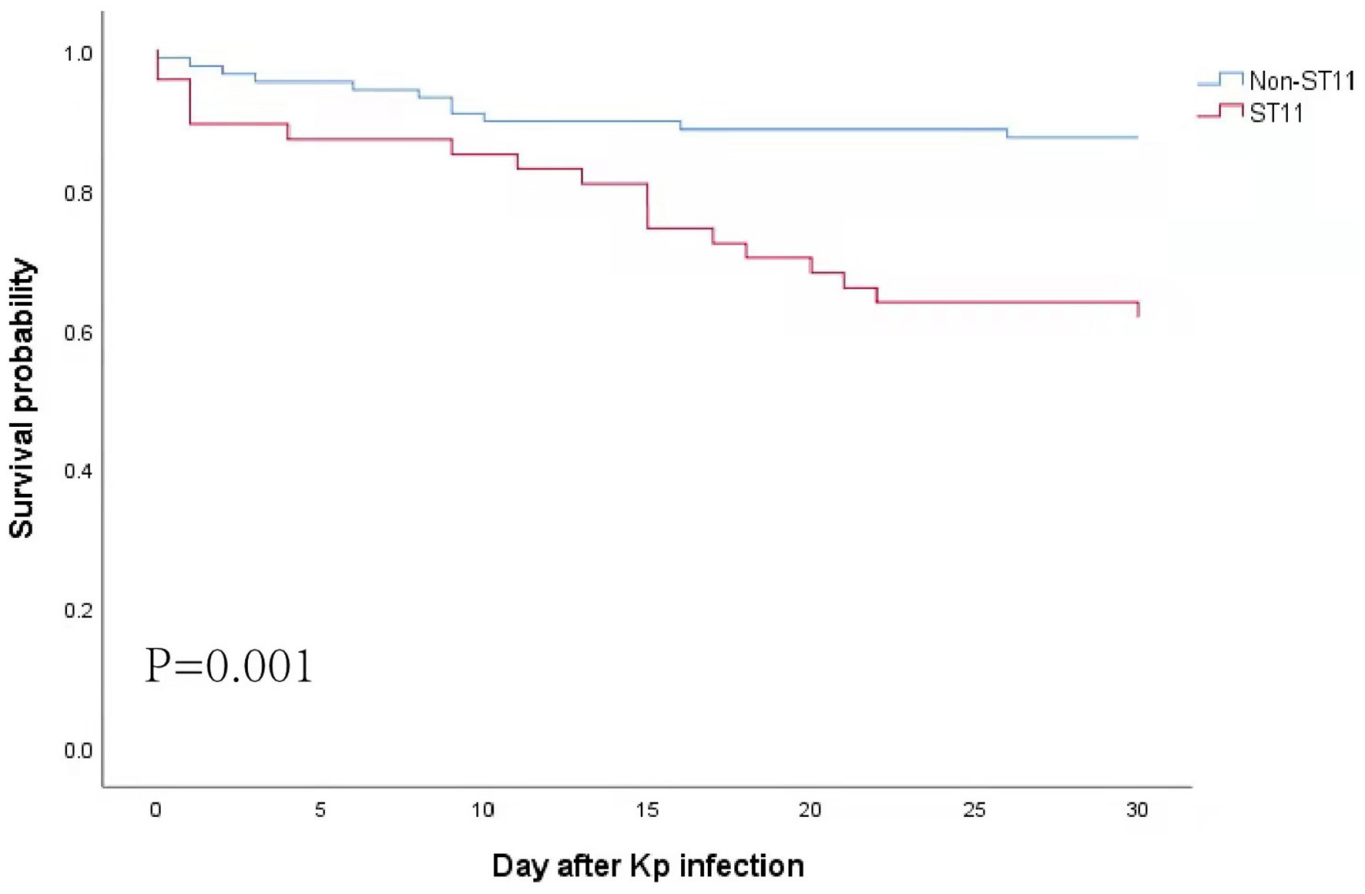
Kaplan–Meier curves for all-cause 30-day mortality. Statistical significance was determined by the log-rank test.

The survival curve revealed that the 30-day mortality rate in patients with ST11 Kp infection was significantly higher than that in patients without ST11 Kp infection (38.3 vs. 12.5%, *P* = 0.001) (Figure 3). Similar results were observed in the hvKp (35.7 vs. 8.9%, *P* = 0.005) and cKp subgroups (42.1 vs. 16.3%, *P* = 0.029) (Supplementary Figure 2). In the ST11 group, the mortality among patients with cKp and hvKp infections was similar (35.7 vs. 42.1%, *P* = 0.644) (Supplementary Figure 3).

Univariate regression analysis showed that ST11 Kp infection [odds ratio (OR) = 4.345] and the CCI (OR = 1.598) were statistically significant risk factors associated with 30-day mortality. In addition, multivariate analysis revealed that ST11 Kp infection appeared to be an independent risk factor for ST11 Kp infection (OR = 2.786) (Table 3).

**Table 3:** Risk factors for death.

**Variable**	**Univariate** **OR^b^ (95% CI^b^)**	**P-value**	**Multivariate** **OR (95% CI)**	**P-value**
ST11 Kp infection	4.345(1.833–10.300)	0.001	2.786(1.089–7.126)	0.032
CCI	1.598(1.206–2.117)	0.001	1.418(1.048–1.918)	0.024

a *OR, Odds ratio*.

b *CI, Confidence interval*.

### Risk Factors for Elevated SOFA Scores in Patients With Kp Infection

The SOFA score was significantly higher in patients with ST11 infection than in the non-ST11 infection patients (6.31 ± 6.04 vs. 2.47 ± 2.97, *P* < 0.001) (Table 1, Supplementary Figure 4). The results of univariate linear analysis revealed that the risk factors for an elevated SOFA score included ST11 Kp infection [risk ratio (RR) = 3.839]) and increased CCI (RR = 0.597). Multivariate analysis showed that compared to non-ST11 infection, ST11 Kp infection caused an increase in SOFA score (RR = 3.579) (Table 4).

**Table 4:** Risk factors for an elevated SOFA score.

**Variable**	**Univariate** **RR^a^ (95% CI^b^)**	***P*** **-value**	**Multivariate** **RR (95% CI^a^)**	***P*** **-value**
ST11 Kp infection	3.839(2.329–5.350)	0.000	3.579(1.906–5.253)	0.000
CCI	0.597(0.149–1.045)	0.009	0.170(-0.297–0.638)	0.472

a *RR, Risk ratio*.

b *CI, Confidence interval*.

## Discussion

The prevalent sequence types of Kp are diverse worldwide. In China, previous studies revealed that ST11 Kp is the main endemic clone, typically presenting as CRKP (7, 28, 29). Similarly, we found that ST11 Kp was highly prevalent in our study. We found that the CCI was significantly higher in patients with ST11 Kp infection than in the non-ST11 infection patients. In addition, our data revealed that the ST11 group was highly associated with catheter usage, ICU admission and mechanical ventilation after Kp detection. It seemed that ST11 Kp-infected patients might have a complicated status. Importantly, the results suggested that all ST11 Kp strains were MDR. Surprisingly, the majority of MDR ST11 isolates were hvKp. The emergence of MDR ST11 hvKp strains threatens the viability of the current therapeutic approach, increasing the severity of infection. The nosocomial dissemination of MDR ST11 hvKp isolates is alarming, and medical staff need to enhance the infection control and management of ST11 strains with “superbug” characteristics and high virulence.

A previous study demonstrated that ST11 Kp was a common class of cKp and was notorious for acquiring various antibiotic resistance genes (30). Notably, the ratio of MDR strains has increased rapidly among ST11 isolates (7, 31). Previous studies reported that ST11 isolates had higher levels of resistance to aztreonam, fosfomycin, amikacin and meropenem than non-ST11 strains (7, 32). Our results also revealed that all ST11 isolates presented with an MDR phenotype, while the drug resistance rate in the non-ST11 group was generally low. Importantly, most MDR-ST11 Kp acquired virulence-associated genes and then evolved into MDR ST11 hvKp strains (18). In terms of the virulence gene spectrum, our data showed that the prevalence of *rmpA2* (42.9%) and *iucA* (59.2%) was very high, and most of the MDR ST11 hvKp possessed *iucA*+*rmpA2*, similar to previous reports, suggesting that they might carry pVir-CR-hvKP4-like virulence plasmids (18, 33, 34). Studies have reported that these strains tested positive on the string test and presented hypervirulence in *Galleria mellonella* infection and human neutrophil models (18, 35). Compared with pLVPK, a 41,231 bp depletion occurred in pVir-CR-hvKP4 that resulted in the loss of the virulence genes *rmpA* and *iro*, but the *iuc* and *rmpA2* genes were retained (18, 34). Further investigations are required to determine whether genetic deletion has a potential effect on the hypervirulence phenotype. Previous studies demonstrated that the K1 and/or K2 capsule serotypes were commonly associated with enhanced virulence (36, 37). However, none of the MDR hvKp strains possessed the K1/K2 serotype in this study. The K47 and K64 serotypes were common within the ST11 group, which is similar to previous studies (38, 39). This result suggested that other genetic elements may play key roles in virulence. A previous study showed that ST11-KL47 was replaced by ST11-KL64 as the endemic subclone (40), which should be further confirmed in a large study. ICE*Kp* represents a key virulence element that exerts a strong influence on the pathogenicity of Kp isolates. ICE*Kp* is responsible for scavenging iron from host transport proteins, thereby enhancing survival and replication within the host (41, 42). Lam et al. (41) reported that *ybt 9* and *ybt 10* were predominant within the ST11 group, and the ICE*Kp3* element was highly associated with *ybt 8* and *ybt 9*. In this study, *ybt9-*ICE*Kp3* was dominant in ST11 isolates, which was similar to a previous study with longitudinal genomic surveillance (43).

As described above, ST11 Kp strains presented all kinds of virulence determinants and showed both MDR and hypervirulent phenotypes, indicating that the prognosis of the patients infected with MDR ST11 hvKp was poor. Gu et al. (18) first reported an outbreak caused by ST11 CR-hvKp in the ICU, and all the patients presented with a poor prognosis. Compared with that of non-ST11 Kp-infected patients (18/20, 90.00%), the 3-year survival rate (28/38, 73.68%) was lower in a report from Liu et al. (21). However, another study reported no significant difference in in-hospital mortality between the ST11 and non-ST11 groups (*P* = 0.795) (22). Remarkably, previous studies have set different endpoints that lead to contradictory conclusions. Too long or too short of a study period results in confounding factors that affect the conclusion. Some studies have indicated that 30-day mortality is a better indicator to analyze the clinical outcomes of infected patients. Of note, the previous study enrolled patients with hospital-acquired pneumonia caused by CRKP, while we enrolled patients with all kinds of Kp infections. Our study highlighted that ST11 Kp infection was significantly associated with higher 30-day mortality than non-ST11 infection. Interestingly, our subgroup analysis revealed that the mortality among patients with cKp and hvKp infections was similar in patients with ST11 strain infection. Notably, the ST11 isolates themselves, not the cKp or hvKp, might be responsible for the poor prognosis. Increased attention should be given to the prevention and control of ST11 Kp infections.

A previous study reported that CRKP appeared to be an independent risk factor for 1-year postoperative mortality in patients after kidney transplantation (44). Additionally, some studies demonstrated that CRKP infection was one of the independent risk factors for death from Kp bloodstream infection (45, 46). However, these studies did not distinguish the specific sequence types that might exert different influences on the mortality of Kp-infected patients. Li et al. observed a high percentage (20/35, 57.1%) of KPC-producing isolates among hvKp strains, in which ST11 strains were dominant (17/35, 48.6%). They found that the KPC-producing isolates were an independent predictor for 30-day mortality of Kp bacteremia patients (47). Similarly, another previous study revealed that ST11 was the most prevalent (66.7%), nearly all of the ST11 isolates were *bla*_KPC_ positive, and *bla*_KPC_ was an independent risk factor for 14-day mortality. It is believed that ST11 strains are the dominant Kp clone in CRKP strains in China, typically carrying *bla*_KPC_ and producing carbapenemase (8, 31). Similarly, all the CRKP isolates were ST11 in our study. In this study, ST11 Kp infection was independently associated with 30-day mortality in Kp-infected patients, indicating that close attention should be given to ST11 strains, not only CRKP.

The SOFA score, a universally recognized indicator to evaluate sepsis, was significantly associated with 30-day mortality in patients with KPC-producing Kp and CRKP infection (48–51). Our study showed that patients with ST11 Kp infection had an elevated SOFA score (RR = 3.579). Moreover, multivariable linear regression revealed that ST11 Kp infection could lead to an increase in the SOFA score, indicating that ST11 Kp strains could cause more serious infections, a higher risk of sepsis and a worse prognosis than non-ST11 Kp strains.

The main limitation of our study is the selection bias and small sample size because it was a retrospective study conducted at a single center. Therefore, further prospective multicenter studies are desirable. Additionally, apart from virulence-associated genes detected by whole-genome sequencing, identification of Kp virulence in *in vitro* and *in vivo* models by using objective evidence, such as *Galleria mellonella*, mouse, or human neutrophil assays, is needed.

In summary, ST11 Kp infection was an independent risk factor for mortality and an elevated SOFA score. Our research demonstrated the notable conclusion that a high prevalence of ST11 Kp strains might be the main cause of high 30-day mortality and SOFA scores in Kp-infected patients. All the ST11 strains presented an MDR phenotype and exhibited great molecularly inferred virulence. For this superbug, it is of great importance to enhance clinical awareness, control and management of ST11 Kp infections.

## Data Availability Statement

The genome sequences in this study were deposited into the China National Center for Bioinformation under BioProject accession no. PRJCA007641. The datasets used and/or analyzed during the current study are available from the corresponding author on reasonable request.

## Ethics Statement

The studies involving human participants were reviewed and approved by Peking University Third Hospital Medical Science Research Ethics Committee. Written informed consent for participation was not required for this study in accordance with the national legislation and the institutional requirements.

## Author Contributions

NS and ML contributed to the study design. NW, ZheW, and JY collected the clinical data. JZ and PY collected the laboratory data and performed the tests. CL and ZhaW analyzed and interpreted the data. ZheW and PY drafted the manuscript. CL revised the manuscript. All authors have reviewed and approved the final version of the manuscript.

## Funding

This study was supported by Beijing Key Clinical Specialty Funding (010071) and by the Clinical Cohort Construction Program of Peking University Third Hospital (BYSYDL2019007).

## Conflict of Interest

The authors declare that the research was conducted in the absence of any commercial or financial relationships that could be construed as a potential conflict of interest.

## Publisher's Note

All claims expressed in this article are solely those of the authors and do not necessarily represent those of their affiliated organizations, or those of the publisher, the editors and the reviewers. Any product that may be evaluated in this article, or claim that may be made by its manufacturer, is not guaranteed or endorsed by the publisher.
