# Supplementary material for: Clinical Outcomes and Microbiological Characteristics of Sequence Type 11 Klebsiella pneumoniae Infection
Source: Front Med (Lausanne). 2022 May 16;9:889020. doi: 10.3389/fmed.2022.889020 (PMC9149164; doi:10.3389/fmed.2022.889020)

Supplementary Material

**Table S2. Clinical Characteristics of ST11 Vs Non-ST11 Kp (hvKp & cKp group).**

| Clinical Characteristics |  | HvKp (n=75) |  |  | cKp (n=64) |  |
| --- | --- | --- | --- | --- | --- | --- |
|  | ST11  n=29 | Non-ST11  n=46 | P *value* | ST11  n=20 | Non-ST11  n=44 | P *value* |
| Basic demographics |  |  |  |  |  |  |
| Age | 79.72±13.008 | 66.78±20.410 | 0.001 | 80.5±11.812 | 70.61±18.021 | 0.029 |
| Male | 16(55.2%) | 30(65.2%) | 0.384 | 13(65.0%) | 31(70.5%) | 0.663 |
| Medical history |  |  |  |  |  |  |
| Diabetes | 11(37.9%) | 18(39.1%) | 0.917 | 9(45.0%) | 12(27.3%) | 0.162 |
| Pulmonary disease | 12(41.4%) | 10(21.7%) | 0.069 | 6(30.0%) | 12(27.3%) | 0.822 |
| Cardiovascular disease | 27(93.1%) | 30(65.2%) | 0.006 | 19(95.0%) | 28(63.6%) | 0.008 |
| Cerebrovascular disease | 19(65.5%) | 14(30.4%) | 0.003 | 12(60.0%) | 19(43.2%) | 0.212 |
| Digestive disease | 7(24.1%) | 17(37.0%) | 0.246 | 8(40.0%) | 17(38.6%) | 0.917 |
| Urinary disease | 14(48.3%) | 15(32.6%) | 0.175 | 11(55.0%) | 11(25.0%) | 0.019 |
| Cancer | 4(13.8%) | 5(10.9%) | 0.988 | 6(30.0%) | 6(13.6%) | 0.227 |
| CCI^a^ | 3.69±1.71 | 2.39±1.48 | 0.026 | 4.30±1.34 | 2.43±1.62 | 0.004 |
| Surgery within 3 months | 2(6.9%) | 4(8.7%) | 1.000 | 1(5.0%) | 4(9.1%) | 0.950 |
| Antibiotics exposure within 90 days | 29(100.0%) | 24(52.2%) | 0.000 | 20(100.0%) | 30(68.2%) | 0.003 |
| Usage of invasive catheters | 29(100.0%) | 22(47.8%) | 0.000 | 20(100.0%) | 28(63.6%) | 0.001 |
| Central intravenous catheter | 18(62.1%) | 6(27.3%) | 0.014 | 15(75.0%) | 7(25.0%) | 0.001 |
| Urinary catheter | 25(86.2%) | 12(54.5%) | 0.012 | 20(100.0%) | 23(82.1%) | 0.129 |
| Endotracheal tube | 9(31.0%) | 2(9.1%) | 0.123 | 7(35.0%) | 5(17.9%) | 0.176 |
| Gastrostomy tube | 25(86.2%) | 10(45.5%) | 0.002 | 19(95.0%) | 20(71.4%) | 0.091 |
| Drainage tube | 7(24.1%) | 8(36.4%) | 0.343 | 6(30.0%) | 3(10.7%) | 0.189 |
| Metastatic infection | 10(34.5%) | 5(10.9%) | 0.013 | 3(15.0%) | 8(18.2%) | 1.000 |
| Infection site |  |  |  |  |  |  |
| Respiratory tract | 17(58.6%) | 26(56.5%) | 0.858 | 15(75.0%) | 21(47.7%) | 0.041 |
| Urine tract | 6(20.7%) | 3(6.5%) | 0.141 | 2(10.0%) | 9(20.5%) | 0.503 |
| Blood | 1(3.4%) | 12(26.1%) | 0.012 | 2(10.0%) | 5(11.4%) | 1.000 |
| Drainage | 1(3.4%) | 2(4.3%) | 1.000 | 0(0.0%) | 1(2.3%) | 1.000 |
| Other | 4(13.8%) | 3(6.5%) | 0.518 | 1(5.0%) | 8(18.2%) | 0.309 |
| Infection type |  |  |  |  |  |  |
| Hospital acquired infection | 29(100.0%) | 32(69.6%) | 0.001 | 20(100.0%) | 35(79.5%) | 0.047 |
| Community acquired infection | 0(0.0%) | 14(30.4%) | 0.001 | 0(0.0%) | 9(20.5%) | 0.047 |
| Laboratory examination |  |  |  |  |  |  |
| Red blood cell | 3.03±0.78 | 3.79±0.90 | 0.000 | 3.04±0.86 | 3.49±0.93 | 0.073 |
| Hemoglobin | 94.31±22.80 | 114.37±23.22 | 0.002 | 94.70±25.83 | 105.80±27.54 | 0.133 |
| White blood cell | 9.74±4.41 | 10.63±4.36 | 0.392 | 10.45±5.63 | 10.33±5.95 | 0.670 |
| Platelet | 177.97±106.80 | 220.67±104.72 | 0.326 | 185.20±158.24 | 215.45±91.62 | 0.433 |
| NEU%^b^ | 76.95±14.42 | 78.92±17.56 | 0.343 | 79.21±17.65 | 78.28±13.85 | 0.557 |
| Total protein | 60.65±7.46 | 62.82±11.43 | 0.347 | 63.45±8.01 | 64.95±8.86 | 0.527 |
| ALbumin | 30.41±3.66 | 32.62±7.16 | 0.085 | 31.31±4.40 | 33.07±4.51 | 0.154 |
| Hematocrit | 1.03±3.94 | 0.35±0.07 | 0.025 | 0.29±0.08 | 0.32±0.08 | 0.206 |
| Vasoactive drugs after Kp detection | 5(17.2%) | 6(13.0%) | 0.869 | 5(25.0%) | 4(9.1%) | 0.191 |
| Admitted in the ICU^c^ | 10(34.5%) | 8(17.4%) | 0.091 | 13(65.0%) | 7(15.9%) | 0.000 |
| Mechanical ventilation after Kp detection | 7(24.1%) | 5(10.9%) | 0.229 | 7(35.0%) | 4(9.1%) | 0.029 |
| SOFA^d^ | 5.28±5.56 | 2.37±3.20 | 0.016 | 7.80±6.53 | 2.57±2.75 | 0.005 |
| 30-day mortality | 10(35.7%) | 4(8.9%) | 0.005 | 8(42.1%) | 7(16.3%) | 0.062 |

^a^CCI, Charlson comorbidity index.

^b^NEU%: Neutrophils percentage

^c^Patients infected kp were then transferred to the ICU.

^d^SOFA, Sequential organ failure assessment.

**Table S3. Clinical Characteristics of hvKp Vs cKp (ST11 group).**

| Clinical Characteristics | ST11 (n=49) | | |
| --- | --- | --- | --- |
|  | HvKp  n=29 | cKp  n=20 | P *value* |
| Basic demographics |  |  |  |
| Age | 79.72±13.01 | 80.5±11.81 | 0.849 |
| Male | 16(55.2%) | 13(65.0%) | 0.491 |
| Underlying medical conditions |  |  |  |
| Diabetes | 11(37.9%) | 9(45.0%) | 0.621 |
| Pulmonary disease | 12(41.4%) | 6(30.0%) | 0.417 |
| Cardiovascular disease | 27(93.1%) | 19(95.0%) | 1.000 |
| Cerebrovascular disease | 19(65.5%) | 12(60.0%) | 0.694 |
| Digestive disease | 7(24.1%) | 8(40.0%) | 0.236 |
| Urinary disease | 14(48.3%) | 11(55.0%) | 0.644 |
| Cancer | 4(13.8%) | 6(30.0%) | 0.306 |
| CCI^a^ | 3.69±1.71 | 4.30±1.34 | 0.592 |
| Surgery within 3 months | 2(6.9%) | 1(5.0%) | 1.000 |
| Antibiotics exposure within 90 days | 29(100.0%) | 20(100.0%) | 1.000 |
| Usage of invasive catheters | 29(100.0%) | 20(100.0%) | 1.000 |
| Central intravenous catheter | 18(62.1%) | 15(75.0%) | 0.343 |
| Urinary catheter | 25(86.2%) | 20(100.0%) | 0.135 |
| Endotracheal tube | 9(31.0%) | 7(35.0%) | 0.771 |
| Gastrostomy tube | 25(86.2%) | 19(95.0%) | 0.604 |
| Drainage tube | 7(24.1%) | 6(30.0%) | 0.648 |
| Metastatic infection | 10(34.5%) | 3(15.0%) | 0.129 |
| Infection site |  |  |  |
| Respiratory tract | 17(58.6%) | 15(75.0%) | 0.236 |
| Urine tract | 6(20.7%) | 2(10.0%) | 0.547 |
| Blood | 1(3.4%) | 2(10.0%) | 0.738 |
| Drainage | 1(3.4%) | 0(0.0%) | 1.000 |
| Other | 4(13.8%) | 1(5.0%) | 0.604 |
| Infection type |  |  |  |
| Hospital acquired infection | 29(100.0%) | 20(100.0%) | 1.000 |
| Community acquired infection | 0(0.0%) | 0(0.0%) | 1.000 |
| Laboratory examination |  |  |  |
| Red blood cell | 3.03±0.78 | 3.04±0.86 | 0.979 |
| Hemoglobin | 94.31±22.80 | 94.70±25.83 | 0.956 |
| White blood cell | 9.74±4.41 | 10.45±5.63 | 0.624 |
| Platelet | 177.97±106.80 | 185.20±158.24 | 0.849 |
| NEU%^b^ | 76.95±14.42 | 79.21±17.65 | 0.553 |
| Total protein | 60.65±7.46 | 63.45±8.01 | 0.219 |
| ALbumin | 30.41±3.66 | 31.31±4.40 | 0.439 |
| Hematocrit | 1.03±3.94 | 0.29±0.08 | 0.948 |
| Vasoactive drugs after Kp detection | 5(17.2%) | 5(25.0%) | 0.763 |
| Admitted in the ICU^c^ | 10(34.5%) | 13(65.0%) | 0.035 |
| Mechanical ventilation after Kp detection | 7(24.1%) | 7(35.0%) | 0.408 |
| SOFA^d^ | 5.28±5.56 | 7.80±6.53 | 0.662 |
| 30-day mortality | 10(35.7%) | 8(42.1%) | 0.658 |

^a^CCI, Charlson comorbidity index.

^b^NEU%: Neutrophils percentage

^c^Patients infected kp were then transferred to the ICU.

^d^SOFA, Sequential organ failure assessment.

Table S4. Genomic characteristics of ST11 Vs Non-ST11 Kp.

|  | ST11(n=49) | Non-ST11(n=90) | P value |
| --- | --- | --- | --- |
| K serotype |  |  |  |
| ***K1*** | **0(0.0%)** | **18(20.0%)** | **0.000** |
| ***K2*** | **0(0.0%)** | **14(15.6%)** | **0.002** |
| *K5* | 0(0.0%) | 3(3.3%) | 0.552 |
| *K20* | 0(0.0%) | 2(2.2%) | 0.540 |
| *K54* | 0(0.0%) | 2(2.2%) | 0.540 |
| *K57* | 0(0.0%) | 3(3.3%) | 0.552 |
| ***K47*** | **26(53.1%)** | **1(1.1%)** | **0.000** |
| ***K64*** | **21(42.9%)** | **1(1.1%)** | **0.000** |
| *K-Unknown* | 2(4.1%) | 5(5.6%) | 1.000 |
| Resistance genes |  |  |  |
| ***aadA2*** | **34(69.4%)** | **11(12.2%)** | **0.000** |
| *aac(3)-IId* | 6(12.2%) | 15(16.7%) | 0.487 |
| *aac(6')-Ib-cr* | 6(12.2%) | 16(17.8%) | 0.393 |
| ***fosA3*** | **45(91.8%)** | **1(1.1%)** | **0.000** |
| *qnrS1* | 13(26.5%) | 12(13.3%) | 0.053 |
| ***mphA*** | **18(36.7%)** | **16(17.8%)** | **0.013** |
| *floR* | 1(2.0%) | 9(10.0%) | 0.164 |
| *arr-3* | 6(12.2%) | 8(8.9%) | 0.739 |
| *sul1* | 7(14.3%) | 16(17.8%) | 0.597 |
| ***tet(A)*** | **21(42.9%)** | **22(24.4%)** | **0.025** |
| ***dfrA1*** | **20(40.8%)** | **4(4.4%)** | **0.000** |
| ***bla_KPC-2_*** | **49(100.0%)** | **8(8.9%)** | **0.000** |
| ***bla_TEM-1D_*** | **46(93.9%)** | **11(12.2%)** | **0.000** |
| *bla_OXA-1_* | 6(12.2%) | 9(10.0%) | 0.684 |
| ***bla_CTX-M-65_*** | **25(51.0%)** | **1(1.1%)** | **0.000** |
| ***bla_SHV-12_*** | **10(20.4%)** | **0(0.0%)** | **0.000** |
| ***bla_SHV-11_*** | **29(59.2%)** | **35(38.9%)** | **0.022** |
| Virulence genes |  |  |  |
| Yersiniabactin |  |  |  |
| ***ybt 9-ICEKp3*** | **49(100.0%)** | **6(6.7%)** | **0.000** |
| ***ybtS*** | **49(100.0%)** | **54(60.0%)** | **0.000** |
| ***ybtX*** | **49(100.0%)** | **54(60.0%)** | **0.000** |
| ***ybtQ*** | **49(100.0%)** | **54(60.0%)** | **0.000** |
| ***ybtP*** | **49(100.0%)** | **54(60.0%)** | **0.000** |
| ***ybtA*** | **49(100.0%)** | **54(60.0%)** | **0.000** |
| ***irp2*** | **49(100.0%)** | **54(60.0%)** | **0.000** |
| ***irp1*** | **49(100.0%)** | **54(60.0%)** | **0.000** |
| ***ybtU*** | **49(100.0%)** | **54(60.0%)** | **0.000** |
| ***ybtT*** | **49(100.0%)** | **54(60.0%)** | **0.000** |
| ***ybtE*** | **49(100.0%)** | **54(60.0%)** | **0.000** |
| Colibactin |  |  |  |
| ***clbL*** | **1(2.0%)** | **22(24.4%)** | **0.001** |
| Aerobactin |  |  |  |
| *iucA* | 29(59.2%) | 39(43.3%) | 0.074 |
| *iucB* | 29(59.2%) | 39(43.3%) | 0.074 |
| *iucC* | 29(59.2%) | 39(43.3%) | 0.074 |
| *iucD* | 29(59.2%) | 39(43.3%) | 0.074 |
| *iutA* | 29(59.2%) | 39(43.3%) | 0.074 |
| Salmochelin |  |  |  |
| ***iroB*** | **0(0.0%)** | **44(48.9%)** | **0.000** |
| ***iroC*** | **0(0.0%)** | **44(48.9%)** | **0.000** |
| ***iroD*** | **0(0.0%)** | **44(48.9%)** | **0.000** |
| ***iroN*** | **0(0.0%)** | **43(47.8%)** | **0.000** |
| Other virulence genes |  |  |  |
| ***rmpA*** | **13(26.5%)** | **40(44.4%)** | **0.038** |
| *rmpA2* | 21(42.9%) | 35(38.9%) | 0.649 |
| ***peg-344*** | **13(26.5%)** | **43(47.8%)** | **0.015** |
| ***peg-589*** | **30(61.2%)** | **37(41.1%)** | **0.023** |
| *iucA+ rmpA2* | 21(42.9%) | 35(38.9%) | 0.649 |
| HvKp | 29(59.2%) | 46(51.1%) | 0.362 |
| **Hypermucoviscosity** | **24(49.0%)** | **62(68.9%)** | **0.021** |

**Figure S1. Department distribution of the ST11 (A) and Non-ST11 (B) Kp infected patients.**


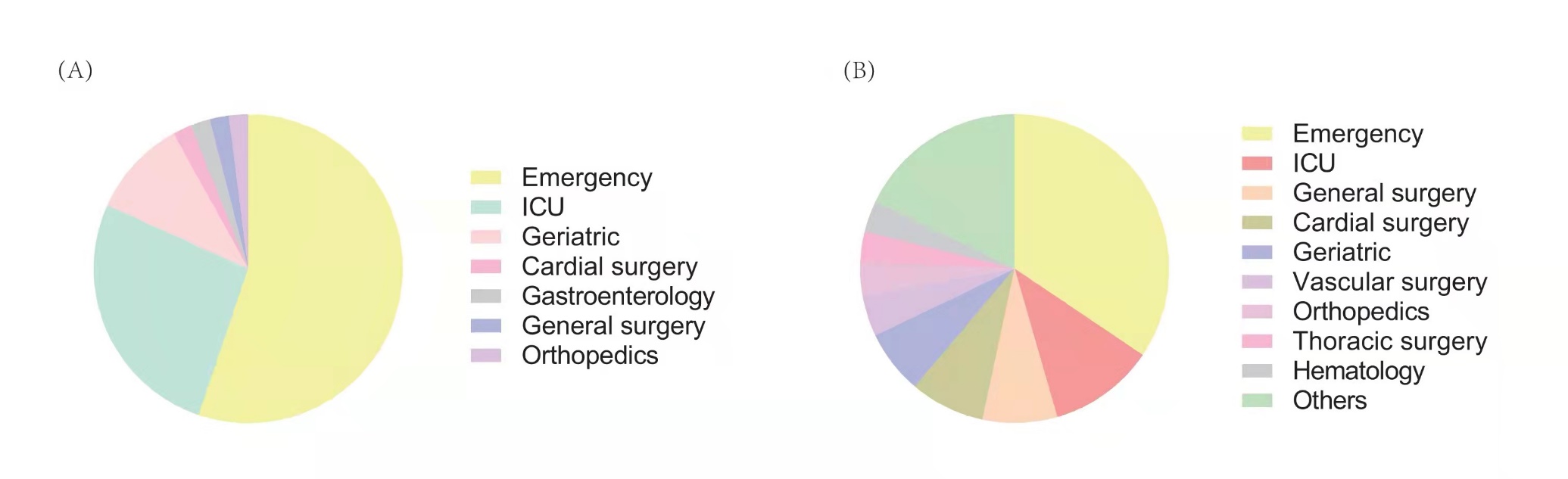


**Figure S2. Kaplan–Meier curves for all-cause 30-day mortality in (A) hvKp and (B) cKp subgroup.**


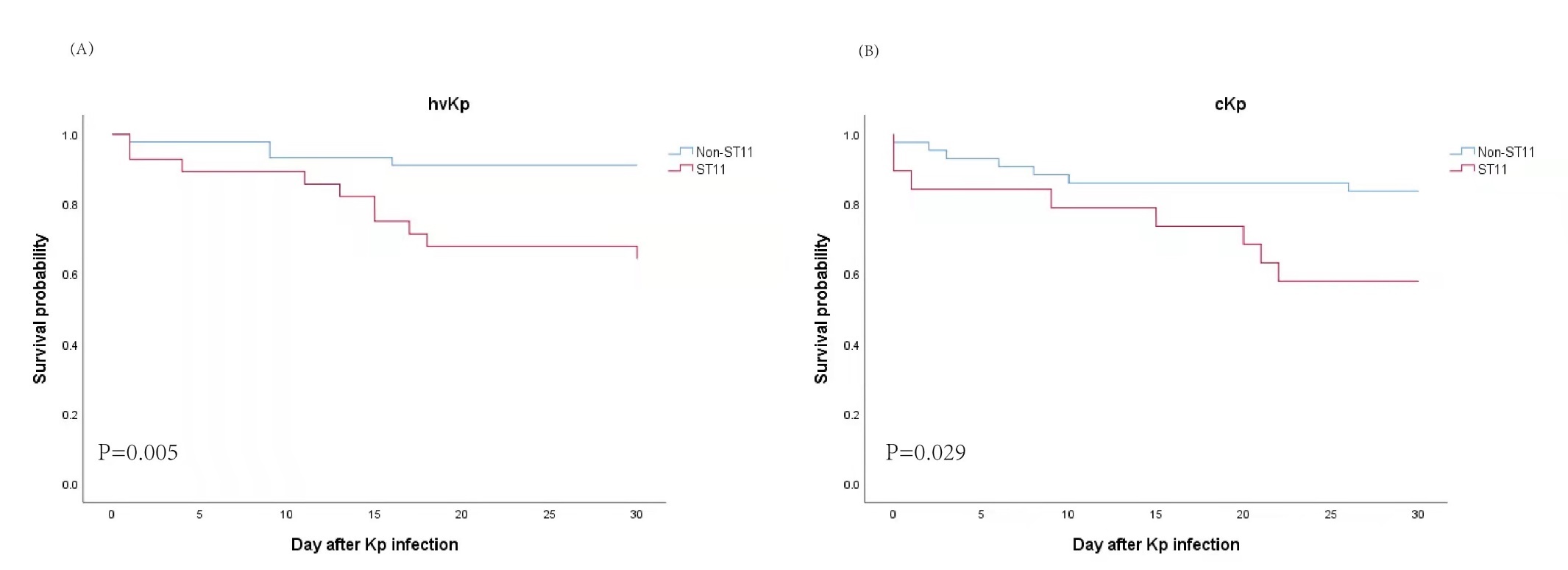


**Figure S3. Kaplan–Meier curves for all-cause 30-day mortality in ST11 Kp group.**


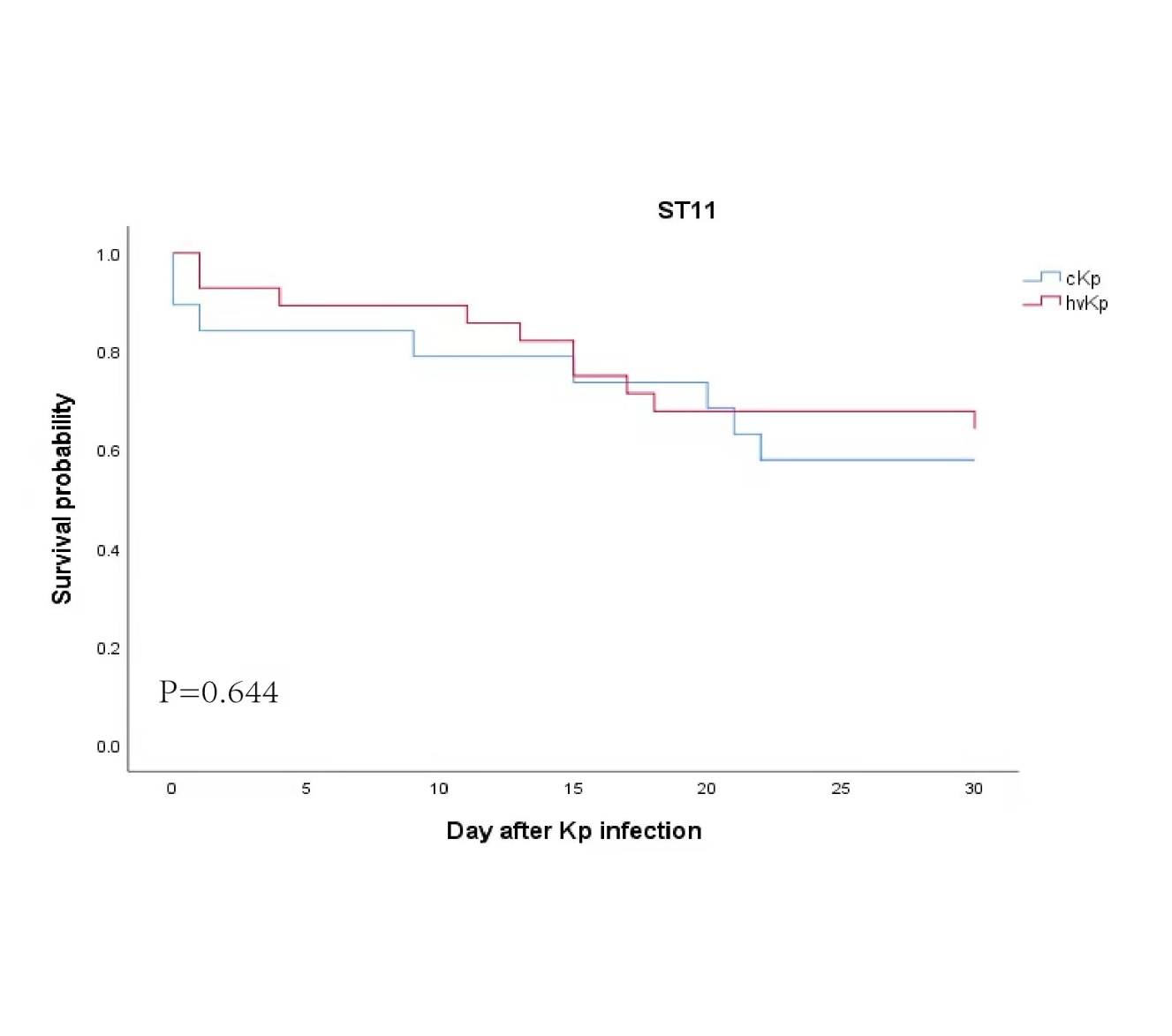


**Figure S4. Box-plot distribution of SOFA score in ST11 vs non-ST11 group.**


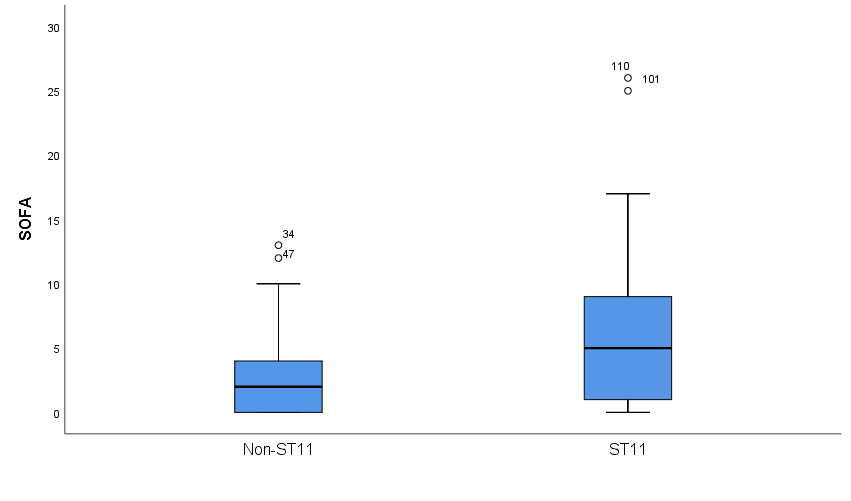

Supplement: Supplementary file 2 [file Data_Sheet_1.docx]
